# The Triglyceride and Glucose Index Is a Predictor of Incident Nonalcoholic Fatty Liver Disease: A Population-Based Cohort Study

**DOI:** 10.1155/2019/5121574

**Published:** 2019-10-07

**Authors:** Aya Kitae, Yoshitaka Hashimoto, Masahide Hamaguchi, Akihiro Obora, Takao Kojima, Michiaki Fukui

**Affiliations:** ^1^Department of Endocrinology and Metabolism, Kyoto Prefectural University of Medicine, Graduate School of Medical Science, Kyoto, Japan; ^2^Department of Gastroenterology, Asahi University Hospital, Gifu, Japan

## Abstract

**Background:**

The triglyceride and glucose index (TyG), defined as the product of triglycerides (TG) and fasting plasma glucose (FPG), is reported as a surrogate index for insulin resistance. Although a cross-sectional study revealed the association between the TyG-index and the prevalence of nonalcoholic fatty liver disease (NAFLD), few studies have investigated the association between the TyG-index and incident NAFLD. Here we investigated whether the TyG-index can be used to predict incident NAFLD.

**Methods:**

This historical cohort study included 16,093 apparently healthy Japanese individuals. The TyG-index was calculated by the established formula: TyG = Ln [TG (mg/dl) ×  FPG (mg/dl)/2]. Fatty liver was diagnosed based on the subjects' abdominal ultrasonography results. We divided the subjects into tertiles according to the levels of TyG-index. Hazard ratios (HRs) of the TyG-index for incident NAFLD were calculated by a Cox proportional hazards regression model.

**Results:**

During the observation period, 27.4% of the men and 11.0% of the women developed NAFLD. The highest TyG-index tertile (men, 8.48 ≤ TyG and women, 7.97 ≤ TyG) (adjusted HR 1.67, 95% CI 1.44–1.94, *p* < 0.001 in the men and 2.06, 1.59–2.70, *p* < 0.001 in the women) and the middle TyG-index tertile (men, 8.00 < TyG ≤ 8.48 and women, 7.53 <TyG ≤7.97) (1.33, 1.15–1.54, *p* < 0.001 in the men and 1.52, 1.16–2.01, *p* < 0.001 in the women) presented a significantly higher risk of incident NAFLD compared to the lowest TyG-index tertile (men, TyG < 8.00 and women, TyG < 7.53).

**Conclusions:**

Our findings demonstrate that the TyG-index is significantly associated with incident NAFLD.

## 1. Introduction

Nonalcoholic fatty liver disease (NAFLD) is associated with insulin resistance through an accumulation of fat in the liver [1]. NAFLD, the prevalence of which is now increasing with lifestyle changes, is not only a risk factor for liver-related morbidity and mortality, but also a risk factor for lifestyle diseases such as type 2 diabetes [2], chronic kidney disease (CKD) [3], and cardiovascular disease [4]. Thus, the early identification of an individual's risk of NAFLD is important for not only the prevention of liver-related morbidity and mortality, but also the prevention of diseases such as type 2 diabetes, CKD, and cardiovascular disease.

The triglyceride and glucose index (TyG), which is defined as the product of an individual's serum levels of triglycerides (TG) and fasting plasma glucose (FPG), has been recommended as a reliable and simple surrogate index for insulin resistance [5–7]. It is reported that TG and FPG are overproduced by a fatty liver [8, 9]. A cross-sectional study revealed an association between the TyG-index and the prevalence of NAFLD [8]. However, there are few studies concerning the association between the TyG-index and incident NAFLD [10]. Zheng et al. [10] revealed that TyG-index is associated with incident NAFLD. However, TyG-index varies by gender; thus, there is a possibility that the effect of TyG-index might differ by gender. Here we performed a longitudinal analysis addressing the question of whether the TyG-index can be used to predict incident NAFLD in a large-scale population of apparently healthy Japanese subjects.

## 2. Materials and Methods

### 2.1. Study Population

We investigated the impact of the TyG-index on incident fatty liver in this historical cohort study of the individuals who underwent a medical health-checkup program at Asahi University Hospital, Gifu, Japan. The characteristics of those individuals and the details of the medical health-checkup program are described elsewhere [11]. Briefly, the results of the checkups were enrolled in a database after the subjects provided informed consent, and their personal identifiable information was shielded. This longitudinal analysis was named NAGALA (NAfld in Gifu Area, Longitudinal Analysis) study.

In the present study, we used the results of the NAGALA subjects who underwent the health-checkup program between 1994 and 2015. The exclusion criteria were as follows: NAFLD at baseline, alcohol intake >30 g/day in men and >20 g/day in women [12], known liver disease, current use of any medication, and missing data of covariants or ultrasonography at baseline examination. For known liver disease, subjects who tested positive for hepatitis B antigen or hepatitis C antibody and those who reported a history of known liver disease (including autoimmune, genetic, viral, and drug-induced liver disease) were also excluded [13]. The Ethics Committee of Asahi University Hospital approved this study.

### 2.2. Data Collection

Lifestyle factors and the medical history of all of the present study's subjects were checked by a standardized self-administered questionnaire. Regarding the subjects' alcohol consumption, we asked the amount and type of alcoholic beverages consumed per week during the prior month, estimating the mean ethanol intake per week; we then calculated the total amount of alcohol consumed per week in grams [14]. Smoking habits were categorized into three groups (never-smoker, past-smoker, and current-smoker). For exercise, the subjects reported the type, duration, and frequency of his or her participation in sports or recreational activities [15]. When a subject regularly performed any type of sports or recreational activities ≥1 × /week, we categorized them as regular exercisers [16].

Each subject's body mass index (BMI) was calculated as body weight in kilograms divided by the square of the subject's height in meters. We performed blood measurements including those of FPG and TG. The TyG-index was calculated with the established formula: TyG = Ln [TG (mg/dl) × FPG (mg/dl)/2] [8].

Diabetes mellitus was defined as HbA1c ≥ 6.5%, fasting plasma glucose ≥126 mg/dl [17] or getting treatment for diabetes.

### 2.3. Definition of Fatty Liver

Fatty liver was diagnosed based on the results of abdominal ultrasonography, which was performed by trained technicians. Gastroenterologists reviewed the ultrasonographic images and made the diagnosis of fatty liver without reference to any of the subject's other individual data. Subjects who showed hepatorenal contrast and liver brightness among four known criteria (hepatorenal echo contrast, liver brightness, vascular blurring, and deep attenuation) were diagnosed as having fatty liver [18].

### 2.4. Statistical Analysis

We investigated the impact of the TyG-index on incident NAFLD separately for men and women. We first divided the subjects into tertiles according to their TyG-index values, and then we compared the baseline characteristics of the subjects. Kolmogorov–Smirnov test was performed to investigate if our data were normally distributed. Continuous variables were analyzed by the Kruskal–Wallis test and Steel–Dwass test and are expressed as the median (interquartile range). Categorical variables were analyzed by Pearson's *Χ*^2^-squared test and are expressed as percentages (numbers).

We performed a Kaplan–Meier analysis for a graphical presentation of the time to the development of NAFLD, and the log-rank test was used to assess differences among groups. To correct for family-wise error, we performed a Bonferroni correction. In the log-rank test, *p* values <0.017 were considered significant.

To investigate the impact of the TyG-index on incident NAFLD, we applied a Cox proportional hazards regression model with a forced entry method. We considered several potential confounders as covariates: baseline gender, age, BMI, alcohol consumption, smoking status, exercise habit, and alanine transaminase. In addition, we also analyzed by gender.

In addition, we also performed receiver operator characteristic (ROC) analyses to calculate area under the ROC curve (AUC) of TyG-index, presence of metabolic syndrome [19] or presence of overweight/obesity, which was defined as BMI ≥ 23 kg/m^2^ [20] for the prediction of developing NAFLD at 2,000 days from baseline examination.

Moreover, it is reported that the triglyceride glucose-body mass index (TyG-BMI-index) is effective in identifying nonalcoholic fatty liver disease [21]. TyG-BMI-index is calculated by TyG-index and BMI. We also considered it as a covariate.

The statistical analyses were performed using the JMP ver. 13.0 software (SAS, Cary, NC), and *p* values <0.05 were considered significant.

## 3. Results

**Figure 1:**
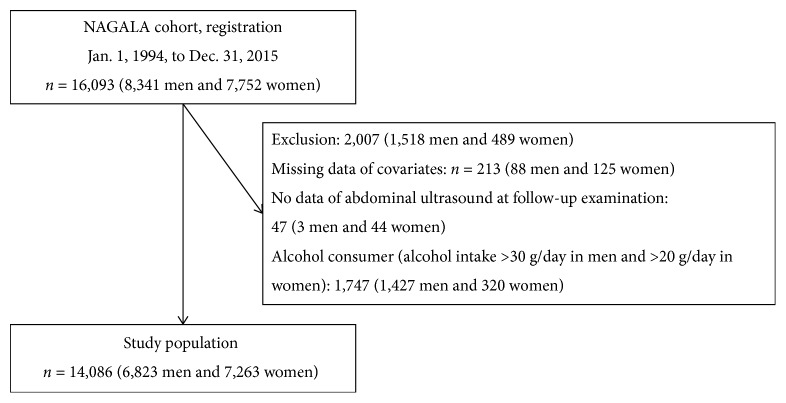
Inclusion and exclusion flow chart. NAGALA: NAfld in Gifu Area, Longitudinal Analysis.

In this study, 16,093 subjects (8,341 men and 7,752 women) who participated in the health checkup program more than two times were extracted from the NAGALA database (Jan. 1, 1994, to Dec. 31, 2015) (Figure 1). Among them, 2,007 subjects (1,518 men and 489 women) were excluded. Therefore, the study population consists of 14,086 subjects (6,823 men and 7,263 women). Median observation periods were 1,881 (interquartile range: 2,771) days in men and 2,198 (interquartile range: 2,645) days in women.

We divided the subjects (males and females separately) into tertiles based on their TyG-index values. The three groups were as follows: for men, the lowest TyG-index tertile (TyG < 8.00), *n* = 2,379; the middle TyG-index tertile (8.00 ≤ TyG < 8.48), *n* = 2,300; and the highest TyG-index tertile (8.48 ≤ TyG), *n* = 2,144; for women, the lowest TyG-index tertile (TyG < 7.53), *n* = 2,468; the middle TyG-index tertile (7.53 ≤ TyG < 7.97), *n* = 2,342; and the highest TyG-index tertile (7.97 ≤ TyG), *n* = 2,453. The clinical and laboratory baseline characteristics of all subjects are provided in Table 1, and the baseline characteristics by gender and each group are provided in Table 2. The baseline metabolic parameters (including BMI, FPG, TG, and blood pressure values) in the highest TyG-index tertile were higher than those in the lowest and middle TyG-index tertiles.

**Figure 2:**
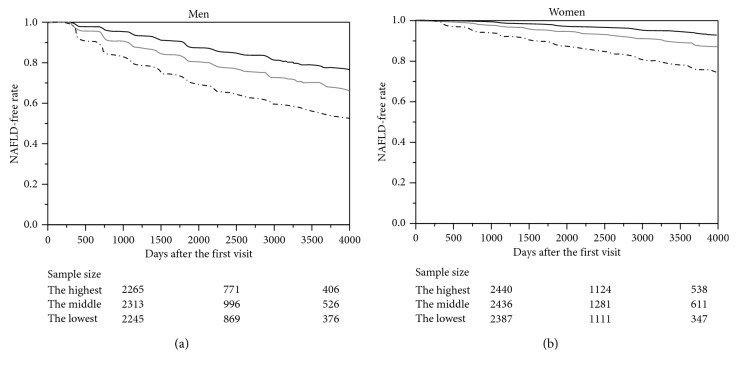
Kaplan–Meier analysis of incident NAFLD-free rate. (a) Men. (b) Women. Black bold line = the lowest TyG-index tertile: TyG <8.00 in the men and TyG <7.53 in the women. Gray line = the middle TyG-index tertile: 8.00 ≤ TyG < 8.48 in the men and 7.53 ≤ TyG < 7.97 in the women. Black dashed line = the highest TyG-index tertile: 8.48 ≤ TyG in the men and 7.97 ≤ TyG in the women. The cumulative incidence of progression among the tertiles showed significant differences (log-rank test, *p* < 0.001 for all three comparisons in both the men and the women). The sample sizes of days 0, 2,000, and 4,000 are shown.

During the observation period, 27.4% (case/*n* = 1,870/6,823) of the men and 11.0% (800/7,263) of the women developed NAFLD. The proportion of incident NAFLD was 4.2% in men and 2.3% in women. According to the Kaplan–Meier analysis (Figure 2), the NAFLD-free rates in the highest and the middle TyG-index tertiles were significantly lower than that in the lowest TyG-index tertile (all *p* < 0.001), and the NAFLD-free rate in the highest TyG-index tertile was significantly lower than that in the middle TyG tertile (both *p* < 0.001) in both the men and the women.

The TyG-index was associated with incident fatty liver (adjusted HR 1.86, 95% CI 1.72–2.01, *p* < 0.001) after adjusting for covariates. The hazard ratios (HRs) for incident fatty liver by gender are shown in Table 3. The highest TyG-index tertile (adjusted HR 2.69, 95% CI 2.35–3.08, *p* < 0.001 in the men and HR 3.94, 95% CI 3.08–5.12, *p* < 0.001 in the women in model 1, adjusting for age; adjusted HR 2.01, 95% CI 1.75–2.31, *p* < 0.001 in the men and HR 2.47, 95% CI 1.92–3.22, *p* < 0.001 in the women in model 2, adjusting for model 1 plus BMI, alcohol consumption, exercise, and smoking; and adjusted HR 1.93, 95% CI 1.68–2.22, *p* < 0.001 in the men and 2.46, 95% CI 1.91–3.21, *p* < 0.001 in the women in model 3, adjusting for model 2 plus alanine transaminase.) had a significantly higher risk of incident NAFLD compared to the lowest TyG-index tertile.

According to the ROC analysis, the AUC of TyG-index (0.64 (95% CI, 0.62–0.65) in men and 0.70 (95% CI, 0.68–0.72) in women) was higher than that of presence of metabolic syndrome (0.53 (95% CI, 0.52–0.54), *p* < 0.001 in men and 0.54 (95% CI, 0.53–0.55), *p* < 0.001 in women) and was tended to be higher than that of presence of overweight/obesity (0.62 (95% CI, 0.61–0.63), *p*=0.113 in men and 0.68 (95% CI, 0.66–0.70), *p*=0.066 in women), although it did not reach a statistical significance.

In addition, the TyG-BMI-index was also associated with incident fatty liver after adjusting for covariates (Δ10 incremental TyG-BMI adjusted HR 1.30, 95% CI 1.28–1.32, *p* < 0.001).

## 4. Discussion

We investigated the association between the TyG-index and incident NAFLD. An earlier study revealed that the TyG-index is associated with incident diabetes [22] and hypertension [23]. Another investigation showed that the TyG-index is associated with the prevalence of NAFLD 87. We demonstrate that a high TyG-index is associated with incident NAFLD even after adjusting for known risk factors in a large-scale population of apparently healthy Japanese subjects. Compared to the past investigation [10], we obtained similar results in a larger prospective study. Furthermore, we conducted analysis by gender. TyG-index differs significantly between men and women, but the association with incident NAFLD was similar.

Possible explanations of the association between the TyG-index and incident NAFLD are as follows: Triglycerides are synthesized from free fatty acids (FFAs), which are produced in the liver [24]. An energy surplus, a decrease of lipolysis in adipose tissue, and an increase of lipogenesis in the liver leads to increased FFAs [24]. When the storage capacity of adipose tissue is limited, the energy surplus—which easily occurs in obesity—leads to an increased efflux of FFAs and an ectopic accumulation of fat in the liver. In addition, insulin resistance suppresses adipose tissue lipolysis [25] and increases de novo lipogenesis [26] through a suppression of insulin ability, although the insulin secretion is increasing. Moreover, high plasma glucose is associated with hyperinsulinemia [27]. In fact, de novo lipogenesis is increased by threefold in patients with NAFLD compared to individuals without NAFLD [24, 26].

Another possible explanation is that the TyG-index is associated with insulin resistance in muscle [28]. An increase in insulin resistance in muscle leads to glucose flow into the liver, which then becomes a fatty liver due to the relatively low insulin resistance in the liver [29]. Taking these previous and present findings together, it is apparent that the TyG-index is associated with incident NAFLD.

The AUC of the TyG-index was higher than that of presence of metabolic syndrome and was tended to be higher than that of presence of overweight/obesity. Considering the results of the Cox proportional hazards regression model, the TyG-index may be related to the development of NAFLD for a different reason than presence of metabolic syndrome and presence of overweight/obesity. Therefore, the TyG-index is expected to be useful as a predictor of incident NAFLD along with these indices.

Moreover, the TyG-BMI-index was also associated with incident fatty liver after adjusting for covariates. Past cross-sectional research has shown that the TyG-BMI-index is related to NAFLD [19]. In our study, even if BMI is used as a covariate, the association between TyG-index and incident NAFLD is recognized. Therefore, the difference between TyG-index and TyG-BMI-index and the proper use of this needs to be further studied in the future.

The limitations of this study should be noted. First, the diagnosis of fatty liver based on ultrasonography findings may be less accurate compared to the diagnosis based on a liver biopsy. However, ultrasonography has also shown high sensitivity and specificity in diagnosing fatty liver [30]. Moreover, the simple use of ultrasonography, without performing a biopsy or magnetic resonance imaging, for NAFLD diagnosis can lead to misclassification of patients because the ultrasonography is reliable and accurate only in detection of moderate-severe fatty liver. Thus, there is a possibility that we have only revealed the significant association between TyG-index and incident moderate-severe steatosis. Second, we did not measure the subjects' insulin concentration directly. However, the purpose of this study was to investigate the association between the TyG-index and incident fatty liver. Third, we included exercise during leisure time as a variable, but we did not have the data of physical activity during working hours. Therefore, we could not conduct a more detailed analysis on the relationship between physical activity and incident NAFLD. Fourth, we asked the amount and type of alcoholic beverages consumed per week during the prior month, estimating the mean ethanol intake per week. This may not accurately reflect the real alcohol consumption during the lifespan. Lastly, the generalizability of our study to non-Japanese population is unclear.

## 5. Conclusions

The TyG-index is associated with incident NAFLD. Considering the potential importance of NAFLD to public health, it is important to check a patient's TyG-index, and if his or her TyG-index is elevated, lifestyle modification is necessary for the prevention of the development of NAFLD.

## Figures and Tables

**Table 1:** Characteristics of the study subjects.

	All
*n*	14086
Age, years	40.0 (13)
Body weight (kg)	57.0 (14.3)
BMI (kg/m^2^)	21.2 (3.4)
Consumption of alcohol (g/week)	1.0 (55.0)
Smoking^†^	
Current-smoker	3134 (22.2%)
Past-smoker	2261 (16.1%)
Never-smoker	8691 (61.7%)
Regular exerciser^†^	2447 (17.4%)
Fasting plasma glucose (mmol/L)	5.1 (0.6)
Hemoglobin A1c (NGSP) (%)	5.1 (0.5)
Triglycerides (mmol/L)	0.7 (0.5)
Total cholesterol (mmol/L)	5.0 (1.1)
HDL cholesterol (mmol/L)	1.5 (0.5)
Aspartate aminotransferase (IU/L)	17.0 (7.0)
Alanine aminotransferase (IU/L)	15.0 (8.0)
Gamma-glutamyltransferase (IU/L)	13.0 (9.0)
Diabetes mellitus^†^	148 (1.1%)
SBP (mmHg)	111.5 (19.0)
DBP (mmHg)	69.5 (12.5)
TyG-index	8.0 (0.8)

The TyG-index was calculated by the following formula: TyG = Ln [TG (mg/dl) × FPG (mg/dl)/2]. Diabetes mellitus: HbA1c ≥ 6.5% or fasting plasma glucose ≥126 mg/dl or getting treatment for diabetes. Continuous variables are presented as median (interquartile range).

**Table 2:** Characteristics of the study subjects analyzed by gender.

Men	All	Lowest TyG-index tertile (TyG < 8.00)	Middle TyG-index tertile (8.00 ≤ TyG < 8.48)	Highest TyG-index tertile (8.48 ≤ TyG)
*n*	6,823	2,379	2,300	2,144
Age, years	40.0 (14.0)	39.0 (11.0)	41.0 (13.0)^*∗*^	43.0 (14.0)^*∗∗∗*^
Body weight (kg)	64.1 (10.9)	62.4 (10.5)	63.8 (0.8)^*∗*^	66.1 (10.8)^*∗∗∗*^
BMI (kg/m^2^)	22.1 (3.2)	21.3 (3.1)	22.0 (3.1)^*∗*^	22.9 (2.9)^*∗∗∗*^
Consumption of alcohol, (g/week)	36.0 (104.0)	18.0 (87.3)	36.0 (89.0)^*∗*^	54.0 (109.0)^*∗∗∗*^
Smoking^†^				
Current-smoker	2,668 (39.1%)	877 (32.9%)	890 (33.4%)	901 (33.8%)
Past-smoker	1,798 (26.4%)	562 (31.3%)	639 (35.3%)	597 (33.2%)
Never-smoker	2,357 (39.1%)	940 (39.9%)	771 (32.7%)	646 (27.4%)
Regular exerciser^†^	1,318 (19.3%)	515 (22.9%)	441 (19.1%)	362 (16.0%)
Fasting plasma glucose (mmol/L)	5.3 (0.6)	5.2 (0.5)	5.3 (0.6)^*∗*^	5.4 (0.6)^*∗∗∗*^
Hemoglobin A1c (NGSP) (%)	5.1 (0.5)	5.1 (0.5)	5.1 (0.5)^*∗*^	5.2 (0.6)^*∗∗∗*^
Triglycerides (mmol/L)	0.9 (0.7)	0.5 (0.2)	0.9 (0.2)^*∗*^	1.5 (0.6)^*∗∗∗*^
Total cholesterol (mmol/L)	5.1 (1.2)	4.7 (1.0)	5.1 (1.0)^*∗*^	5.4 (1.1)^*∗∗∗*^
HDL cholesterol (mmol/L)	1.3 (0.4)	1.5 (40.5)	1.3 (0.4)^*∗∗∗*^	1.1 (0.4)^*∗∗∗*^
Aspartate aminotransferase (IU/L)	18.0 (7.0)	17.0 (7.0)	17.0 (7.0)	18.0 (6.0)^*∗∗∗*^
Alanine aminotransferase (IU/L)	18.0 (10.0)	16.0 (8.0)	18.0 (10.0)^*∗*^	20.0 (10.5)^*∗∗∗*^
Gamma-glutamyltransferase (IU/L)	17.0 (12.0)	15.0 (8.0)	17.0 (10.0)^*∗*^	21.0 (18.0)^*∗∗∗*^
Diabetes mellitus^†^	118.0 (1.7%)	6.0 (0.3%)	26.0 (1.1%)	86.0 (3.8%)
SBP (mmHg)	115.5 (17.5)	113.0 (16.5)	115.0 (18.0)^*∗*^	118.0 (18.0)^*∗∗∗*^
DBP (mmHg)	72.5 (12.5)	70.5 (12.0)	72.5 (12.0)^*∗*^	74.5 (12.5)^*∗∗∗*^
TyG-index	8.2 (0.7)	7.7 (0.4)	8.2 (0.3)^*∗*^	8.8 (0.4)^*∗∗∗*^

Women	All	Lowest TyG-index tertile (TyG < 7.53)	Middle TyG-index tertile (7.53 ≤ TyG < 7.97)	Highest TyG-index tertile (7.97 ≤ TyG)

*n*	7,263	2,468	2,342	2,453
Age, years	40.0 (11.0)	38.0 (8.0)	40.0 (11.0)^*∗*^	44.0 (3.0)^*∗∗∗*^
Body weight (kg)	51.2 (8.7)	49.9 (7.9)	51.0 (8.4)^*∗*^	52.7 (9.3)^*∗∗∗*^
BMI (kg/m^2^)	20.4 (3.2)	19.7 (2.7)	20.3 (3.0)^*∗*^	21.2 (3.4)^*∗∗∗*^
Consumption of alcohol (g/week)	0.0 (12.0)	1.0 (4.2)	0.0 (12.0)	0.0 (12.0)
Smoking				
Current-smoker	466 (6.4%)	139 (29.8%)	151 (32.4%)	176 (37.8%)
Past-smoker	463 (6.4%)	169 (36.5%)	142 (30.6%)	152 (32.8%)
Never-smoker	6,334 (87.2%)	2,160 (34.1%)	2,049 (32.5%)	2,125 (33.6%)
Regular exerciser^†^	1,129 (15.5%)	327 (13.7%)	406 (16.7%)	396 (16.2%)
Fasting plasma glucose (mmol/L)	4.9 (0.5)	4.8 (0.5)	4.9 (0.5)^*∗*^	5.1 (0.6)^*∗∗∗*^
Hemoglobin A1c (NGSP) (%)	5.1 (0.5)	5.1 (0.5)	5.1 (0.5)^*∗*^	5.2 (0.5)^*∗∗∗*^
Triglycerides (mmol/L)	0.6 (0.4)	0.4 (0.1)	0.6 (0.2)^*∗*^	0.9 (0.4)^*∗∗∗*^
Total cholesterol (mmol/L)	5.0 (1.2)	4.6 (0.9)	5.0 (1.2)^*∗*^	5.4 (1.2)^*∗∗∗*^
HDL cholesterol (mmol/L)	1.6 (0.5)	1.7 (0.5)	1.6 (0.7)^*∗*^	1.5 (0.5)^*∗∗∗*^
Aspartate aminotransferase (IU/L)	16.0 (6.0)	16.0 (6.0)	15.0 (6.0)^*∗*^	16.0 (6.0)^*∗∗*^
Alanine aminotransferase (IU/L)	13.0 (6.0)	13.0 (6.0)	13.0 (6.0)	14.0 (6.0)^*∗∗∗*^
Gamma-glutamyltransferase (IU/L)	11.0 (5.0)	11.0 (4.0)	11.0 (5.0)^*∗*^	11.0 (6.0)^*∗∗∗*^
Diabetes mellitus^†^	30 (0.4%)	0 (0%)	2 (0.1%)	28 (1.2%)
SBP (mmHg)	107.0 (17.5)	104.0 (15.5)	106.0 (16.5)^*∗*^	111.5 (20.0)^*∗∗∗*^
DBP (mmHg)	66.5 (12.0)	64.5 (11.0)	65.5 (11.4)^*∗*^	69.5 (13.0)^*∗∗∗*^
TyG-index	7.8 (0.7)	7.3 (0.4)	7.8 (0.3)^*∗*^	8.3 (0.4)^*∗∗∗*^

The TyG-index was calculated by the following formula: TyG = Ln [TG (mg/dl) × FPG (mg/dl)/2]. Diabetes mellitus: HbA1c ≥ 6.5% or fasting plasma glucose ≥126 mg/dl or getting treatment for diabetes. Continuous variables are presented as median (interquartile range). The differences among tertiles were evaluated by one-way ANOVA and Tukey's HSD test or the Kruskal–Wallis test and Steel–Dwass test. ^*∗*^*p* < 0.05 versus the lowest TyG-index tertile; ^*∗∗*^*p* < 0.05 versus the middle TyG-index tertile. Categorical variables are expressed as number (%), and the differences among tertiles were evaluated by Pearson's Χ2 test. ^†^*p* < 0.05.

**Table 3:** Hazard ratios of incident NAFLD.

Men	Model 1	*p*	Model 2	*p*	Model 3	*p*
Age, years	1.00 (1.00–1.01)	0.319	1.01 (1.00–1.01)	0.047	1.01 (1.00–1.01)	0.007
TyG-index tertiles:						
Highest (8.48 ≤ TyG)	2.69 (2.35–3.08)	<0.001	2.01 (1.75–2.31)	<0.001	1.93 (1.68–2.22)	<0.001
Middle (8.00 ≤ TyG < 8.48)	1.64 (1.42–1.89)	<0.001	1.44 (1.25–1.67)	<0.001	1.42 (1.23–1.64)	<0.001
Lowest (TyG < 8.00)	Ref	–	Ref	–	Ref	—
BMI (kg/m^2^)	–	–	1.22 (1.20–1.24)	<0.001	1.21 (1.18–1.23)	<0.001
Log (alcohol consumption + 1)	–	–	0.91 (0.89–0.93)	<0.001	0.91 (0.89–0.93)	<0.001
Exercise, yes	–	–	0.77 (0.67–0.88)	<0.001	0.78 (0.68–0.89)	<0.001
Past-smoker	–	–	0.98 (0.86–1.11)	0.757	0.98 (0.86–1.11)	0.734
Current-smoker	–	–	0.92 (0.82–1.03)	0.142	0.92 (0.82–1.03)	0.139
Alanine transaminase (IU/L)	–	–	–	–	1.01 (1.01–1.01)	<0.001

Women	Model 1	*p*	Model 2	*p*	Model 3	*p*

Age, years	1.03 (1.02–1.04)	<0.001	1.03 (1.02–1.04)	<0.001	1.03 (1.02–1.04)	<0.001
TyG-index tertiles:						
Highest (7.97 ≤ TyG)	3.94 (3.08–5.12)	<0.001	2.47 (1.92–3.22)	<0.001	2.46 (1.91–3.21)	<0.001
Middle (7.53 ≤ TyG < 7.97)	1.95 (1.49–2.57)	<0.001	1.59 (1.22–2.10)	<0.001	1.59 (1.22–2.10)	<0.001
Lowest (TyG < 7.53)	Ref	–	Ref	–	Ref	–
BMI (kg/m^2^)	–	–	1.32 (1.29–1.35)	<0.001	1.32 (1.29–1.35)	<0.001
Log (alcohol consumption + 1)	–	–	0.94 (0.89–0.99)	0.013	0.94 (0.89–0.99)	0.013
Exercise, yes	–	–	0.92 (0.73–1.13)	0.426	0.92 (0.73–1.13)	0.436
Past-smoker	–	–	0.93 (0.62–1.32)	0.686	0.93 (0.62–1.32)	0.685
Current-smoker	–	–	1.13 (0.83–1.51)	0.405	1.14 (0.83–1.51)	0.406
Alanine transaminase (IU/L)	–	–	–	–	1.00 (1.00–1.01)	0.099

The analysis of past and current smokers used nonsmoker as the reference. The analysis of the TyG-index used the lowest TyG-index tertile as the reference.

## Data Availability

The data used to support the findings of this study are available from the corresponding author upon request.
